# Digitalizing Specialist Smoking Cessation Support in Pregnancy: Views of Pregnant Smokers

**DOI:** 10.1093/ntr/ntae184

**Published:** 2024-07-26

**Authors:** Pippa Belderson, Lisa McDaid, Joanne Emery, Tim Coleman, Jo Leonardi-Bee, Felix Naughton

**Affiliations:** School of Health Sciences, University of East Anglia, Norwich, UK; School of Health Sciences, University of East Anglia, Norwich, UK; School of Health Sciences, University of East Anglia, Norwich, UK; Centre for Academic Primary Care, School of Medicine, University of Nottingham, Nottingham, UK; Centre for Evidence Based Healthcare, School of Medicine, University of Nottingham, Nottingham, UK; School of Health Sciences, University of East Anglia, Norwich, UK

## Abstract

**Introduction:**

Unsupported attempts to quit smoking during pregnancy have a low success rate. Chances of quitting successfully are higher with an interpersonal treatment program but there is low uptake of this in the United Kingdom. Delivering a pregnancy-specific treatment program digitally may provide an alternative treatment route. This study explored pregnant smokers’ perceptions of barriers and facilitators to using digital cessation support, along with identifying modes of delivery and engagement enhancers.

**Aims and Methods:**

Semi-structured interviews were carried out with an ethnically and socioeconomically diverse sample of 25 participants with recent experience of attempting to quit smoking in pregnancy, aged 20–40, from the United Kingdom. An inductive thematic analysis approach was used.

**Results:**

Digital smoking cessation support, particularly a smartphone app, for pregnancy was felt to overcome many barriers to engaging with interpersonal support, being viewed as more convenient, and nonjudgmental, providing better consistency of advice, and enhancing privacy and autonomy. However, some participants felt that removing access to a human could undermine a digital support package and reduce engagement. Popular engagement enhancers included self-monitoring (eg, digital recording of smoking; smartphone-linked carbon monoxide monitoring), online communities, and remote access to nicotine substitution options. Digital support was viewed as having potential as a stand-alone intervention or working in conjunction with standard interpersonal treatment.

**Conclusions:**

The findings support the investigation of a digital support package as both a stand-alone and adjunct to standard interpersonal cessation support in pregnancy to increase the proportion of pregnant smokers who make a supported quit attempt.

**Implications:**

In many countries like the United Kingdom, there are few smoking cessation options routinely available that provide effective support for smoking cessation in pregnancy. To maximize impact, health services need an effective range of strategies to engage with and support quit attempts made by all pregnant smokers, particularly as interpersonal support options are not often well used. Development of a pregnancy-specific digital support package for smoking cessation in pregnancy may represent a means to help address this gap.

## Introduction

Smoking in pregnancy remains one of the most important modifiable causes of adverse pregnancy outcomes. Extensive evidence shows that tobacco use in pregnancy is associated with higher rates of preterm birth, stillbirth, miscarriage, and low birth weight.^1^ Babies born to people who smoke in pregnancy are also more likely to face breathing difficulties and other health problems in later life.^2^ Moreover, people living in the most deprived areas are six times more likely than the those in least deprived areas to smoke during pregnancy, reinforcing health inequalities^3^Intensive behavioral counseling is an effective treatment for smoking cessation in pregnancy.^4^ This is usually offered in conjunction with other behavioral support strategies such as social support, incentives, and biochemical feedback, that is, carbon monoxide (CO) breath test^4^ and, in a small number of high-income countries, nicotine replacement therapy (NRT).,^5,6^ In the United Kingdom, specialist cessation support for pregnancy is available through the National Health Services (NHS). Known as the “Standard Treatment Program” (STP),^7^ this pregnancy-specific support combines weekly interpersonal behavioral counseling sessions (either in person or by phone) with CO testing and access to free NRT. An evidence synthesis found four UK studies which have investigated STP outcomes; reported 4-week quit rates were found to be between 32% and 8%,.^8^ Despite reports of relatively high interest in accessing support to quit smoking in pregnancy^9^ uptake of NHS specialist cessation support is estimated to be 12%–18%.,^9,10^ Although specialist cessation support is available nationally^10^ the low level of uptake highlights a gap between interest and engagement.^9^ Interestingly, the study by Naughton et al.^9^ found that likelihood of using NHS cessation support in pregnancy was associated with interest in support, having previously tried to quit smoking in pregnancy and older age, but no other demographic factors were significant.

One explanation for low uptake may be implementation issues with the recommended treatment pathway, such as how NHS specialist support is promoted,^11^ a lack of integration between services^12^ and local variability in delivery models.^13^ The new NHS Long Term Plan smoke-free pregnancy pathway, where all pregnant smokers should be offered specialist support within, rather than outside, the maternity setting, has been designed to help overcome these barriers.^14^ However, individual barriers to accessing interpersonal cessation support remain, such as fear of stigmatisation^12,15,16^ or feeling pressured by health professionals,^12,13^ not having the time to engage and finding services inflexible in terms of the treatment offered.^12,17^

UK National Institute for Health and Care Excellence (NICE) guidance recommends the provision of self-help support, including digital, for pregnant smokers who are reluctant to engage with a specialist advisor,^6^ but currently no such routine support is available. NHS digital cessation support exists but does not have a specific focus on pregnancy, even though this is often the main reason why people want to quit.^18^ Pregnancy-orientated digital cessation interventions, including text messaging, websites and apps,^19–24^ show promise as low-cost cessation interventions.^23^ However, no existing digital interventions directly address the, arguably, most important modifiable risk factor for failure to quit—tobacco dependence.^25,26^ Incorporating the remote provision of NRT, and potentially electronic cigarettes (e-cigarettes) if these become standard care for smoking cessation in pregnancy, could strengthen the effectiveness of digital support. Research has also highlighted that embedding strategies to optimize engagement with digital interventions is crucial.^19^ Furthermore, concerns have been expressed about whether UK pregnant smokers who do not engage with routine interpersonal support would be motivated to engage with self-directed support, as reasons for low engagement extend beyond access.^12^

Understanding whether pregnant smokers are interested in digital cessation support and generating ideas about core components are important for guiding future interventions that would be acceptable to, and used by, this population. Qualitative research in this area is limited and existing work predates the COVID pandemic,^27^ after which there has been an accelerated shift towards digitalization. In this study we sought to explore potential barriers and facilitators to a digital support package for smoking cessation in pregnancy, along with potential modes of delivery and engagement enhancers.

## Methods

This pragmatic qualitative study was approved by Research Ethics Committees at the University of East Anglia (Project ID- ETH2223-0925) and is reported following COREQ guidelines.^28^

## Recruitment

A combination of promotion via social media and paid-for Facebook advertising was used for recruitment. The adverts contained a link to an online registration page. Potential participants were sent an introductory email and participant information sheet, followed by screening for eligibility. Eligibility criteria were being aged 16 years or over, living in the United Kingdom, pregnant or less than 12 months postpartum, and attempting to stop smoking on at least one occasion while pregnant (either successfully or unsuccessfully). Recruitment ceased when we were satisfied that we reached data saturation, in line with a reflexive thematic analysis approach, when no distinctly different themes were arising in interviews.^29^ Full consent was obtained prior to interview, and a £20 shopping voucher was offered as compensation.

## Interviews

Semi-structured interviews were conducted via phone or video call, by two female qualitative researchers (PB and LM) experienced in smoking cessation research. Interview time ranged from 47 minutes to 1½ hours. Interviews were conducted between October 2022 and March 2023. The topic guide and participant materials were informed by findings from our parallel work with smoking cessation experts^30^ and working closely with three public partners. The work with smoking cessation experts involved focus groups and interviews with participants from academic, policy and practitioner backgrounds. Findings informed development of the interview topic guide content, allowing us to refine questions and probes to further explore key themes from the pregnant person’s perspective. This guided particular interview attention on relational aspects of support, accountability, and anonymity; engagement enhancers such as remote CO monitoring or nicotine substitution; and potential pathways for implementation. In addition three female public partners who were all “experts by experience,” having previously smoked tobacco in pregnancy, helped advise on appropriate content and language for the topic guide and the accompanying illustrative prompt sheet. Our use of the terminology “pregnant smokers” in this paper follows consultation with our public partners, who preferred this phrase.

The topic guide covered: (1) quitting smoking and digital cessation support experience (2) potential digital engagement enhancers, and (3) initiating and structuring digital support (Supplementary Materials 1). To stimulate discussion, we provided participants with a prompt sheet illustrating different examples of digital smoking cessation support to reflect on in advance of the interview (Supplementary Materials 2). All interviews were recorded and fully transcribed verbatim. Field notes were used to record initial themes and researcher reflections.

## Analysis

We undertook thematic analysis of interview data,^31^ using NVivo V12 to support analysis. Initial inductive coding of 10 transcripts was conducted by PB, with two of the same transcripts independently coded by LM. Following this initial inductive analysis, we developed a broad coding frame, agreed by consensus with the research team (PB, LM, and FN). One researcher (PB) applied this coding frame to all subsequent transcripts, while retaining flexibility to add additional inductive codes when appropriate.

## Findings

### Sample

The purposive sample comprised 25 participants drawn from 70 expressions of interest. Participants came from a wide range of localities across England, including major metropolitan and rural areas (Table 1). As an indicator of socioeconomic status we applied the Index of Multiple Deprivation, which is based on postcode and is the official, small-area-based measure of relative deprivation in England.^32^ This showed that participants were drawn from across quintiles of deprivation. Mean age was 30 years (range 20–40 years) and the sample included participants from a diversity of ethnic backgrounds. A total of 16 (64%) of the sample had accessed the STP interpersonal support for smoking cessation while pregnant. Of these, 10 had quit successfully and 6 were smoking at the time of interview.

**Table 1.** Summary Characteristics of Interview Sample

Characteristic	Frequency (%) *N* = 25
Age (years)	Mean 30 (range 20-40)
*Ethnicity*	
Asian Pakistani	1 (7%)
Black British	3 (12%)
Black African	1 (7%)
Black Other	1 (7%)
White British	17 (68%)
White Other (Eastern European)	1 (7%)
*Index of multiple deprivation quintiles* ^*^ *(1 = most deprived)*	*(N = 24)*
1	3 (13%)
2	5 (21%)
3	9 (38%)
4	6 (25%)
5	1 (4%)
*Rural/Urban classification (ONS, 2023)*	
England A1: Within a very large conurbation	7 (28%)
England C1: A town or city, surrounded by populated countryside	7 (28%)
England C2: A town or city, surrounded by sparsely inhabited countryside	2 (8%)
England D1: a small town or larger village	5 (20%)
England E1: a rural village	2 (8%)
England F1: a rural hamlet or isolated dwellings	1 (4%)
Currently pregnant postpartum	19 (76%) (mean gestation = 24 weeks, range: 7–36 weeks) 6 (24%) (mean = 17 weeks, range: 1 week–6 months)
Smoking status in pregnancy	
Smoking, with quit attempt/s	14 (56%)
Successful quit	11 (44%)
Used/using vape in pregnancy	11 (44%)
Used/using NRT in pregnancy	6 (24%)
Accessed STP interpersonal support for smoking cessation in pregnancy	16 (64%)
Downloaded or used a generic stop smoking app	10 (40%)
Partner a current smoker	14 (56%)

^*^The Index of Multiple Deprivation (IMD) is the official measure used in England to classify the relative deprivation of small areas, based on postcode.^32^.

### Interview Findings

Four key themes from the analysis were identified: (1) Receptivity and experience, (2) Machine versus human, (3) Engagement enhancers, and (4) Initiation, implementation and integration.

### Receptivity and Experience

Interviewees expressed high receptivity to the idea of a digital support package and comfort with the digital space. In response to the question “Imagine you were offered the option of a digital stop smoking support package or practitioner stop smoking support, which would you choose?” participants, including those who had accessed interpersonal support, largely expressed a (hypothetical) preference for digital modes,

A smartphone app was seen as preferable, as it offers an accessible, centralized source of support:

*“I use my phone for everything so an app would probably be quite useful...I think it’s just having everything in one place.”* [IV04, postpartum, abstinent]

Many participants used pregnancy apps, but there was little experience or recall of content or support around smoking cessation within these, and relatively low awareness of digital support options for smoking cessation: “I didn’t even know there was such a thing as stop smoking apps, otherwise I would have tried it... yeah, a thousand percent.” [IV25 pregnant, abstinent]. Some described having looked online but feeling unsure about where to obtain reliable information and support, and a need for direction to a trustworthy source of support was expressed:

*“Websites are all contrary so like some of them say different things …if you get recommended an app to go on, all the information is there and you’re on the right app, you’re not going to the wrong website.”* [IVO6 pregnant, smoking]

Around one third of participants had explored stop smoking apps, some having been signposted to them by their local stop smoking service. Levels of use varied. Participants indicated they would have felt more invested in engaging with this kind of app if it was tailored for pregnancy:

“*I feel like that makes it feel a lot more relatable and it’s more likely that someone will pick that app over like the NHS Quit Smoking app because they feel like the information would still be a bit more tailored to them.”* [IV24 pregnant, abstinent]

### Machine Versus Human

Key themes around the comparative advantages of digital support and standard interpersonal support are presented below.

### “Machine”: Perceived Advantages of Digital Support

#### Stigmatization and Interaction Preferences

Stigma and fear of judgment from healthcare professionals (HCPs) was often raised as a barrier to engaging with interpersonal support or honest self-reporting of smoking behavior. Some felt that interpersonal discussions around smoking could damage their relationship with their midwife, and hesitance about accessing or continuing to engage with interpersonal cessation support could lead to disengagement:

“*The first phone call was ‘Well you’re killing your baby, this that and the other’ and straightaway it just, I just couldn’t ring them or speak to them again.”* [IV22 postpartum, smoking]

These feelings were sometimes described even when the HCP was perceived as supportive and nonjudgmental. Digital support appealed to those who found in-person interaction challenging or were experiencing anxiety:

*“I’d choose the app version merely because I personally suffer with agoraphobia and anxiety so for me to be able to do it in the comfort of my own home would have been a much more positive experience because of the stress of having to attend appointments.”* [IV18 pregnant, smoking]

The anonymity of a digital option was also be felt to be more conducive to honest self-reporting, as one participant noted: “*I think it’s easier to tell an app if you’ve had a craving or if you’ve smoked one fag in a day than it is to tell a human being.”* [IV14 pregnant, abstinent].

#### Autonomy

Some participants had found services inflexible in terms of treatment options offered, and felt the comparative autonomy and control that a digital package may offer was appealing:

*“Digitally I would say you could tailor your own experience the way you like it I guess or the way you feel like you needed support with, whereas with an Advisor they very much have their set way.”* [IV07 pregnant, abstinent]

Some participants suggested that a trusted digital support package could also play a role in overcoming inconsistencies in advice received from HCPs by providing *“reliability and kind of consistency*” [IV24 pregnant, abstinent].

#### Convenience and Continuity of Access

Participants discussed how juggling a young family and work commitments impacted their ability to engage with interpersonal cessation support. Digital support was regarded as highly convenient by comparison.

*“I know it’s only a five minute chat but you have to kind of wait around for them to ring so I just quickly say ‘Yeah I’m doing really well, speak to you next week’ just to get her off. Whereas if you had that app and actually you can do it at any time of the day, when you sit down of an evening and you think ‘Oh actually no I have struggled today so I can put that in on my app and get some support for it’, it’s just more suited for you then isn’t it.”* [IV13 pregnant, abstinent]

The idea of ‘24/7’ availability was consistently highlighted in interviews as positive: “I think having a digital app - it’s more with you every day, it’s with you all the time so it’s more accessible.” [IV25 pregnant, abstinent]. Continuous availability was particularly relevant to evenings or night, when some participants described feeling very alone and in need of support. The intermittent nature of standard interpersonal support had left some feeling adrift at times (one relapsed when her advisor was on leave):

*“I think it would definitely support with motivation on like a daily basis whereas if you’ve got a gap in-between talking to your advisor.”* [IV07 pregnant, abstinent]

The following excerpt exemplifies how all the themes outlined above could interact and play out for one individual (who was accessing the STP but still smoking), to hinder perceived effectiveness interpersonal support:

*Almost every single appointment [with stop smoking adviser] I’ve found is “do you want to know what it’s doing to your baby” and ‘you might hurt your baby blah blah blah’ but what it does to me it damages the relationship I have with them . . . because I feel so forced. Whereas [with digital support] I have a choice whether I click on it . . . I could click in and out of the hard to read stuff like when I feel I’m ready.... then I don’t have to worry about my kids when they go wild at an appointment, I’m a private person so it would be a lot easier and I would probably be more inclined to look at it . . . opening an app you can kind of type out how you’re feeling rather than having to go and meet my advisor and be like ‘I’m doing really well and so I’ve not had cravings all day, I’ve not had a cigarette all day, I feel fine’ but then later that evening when everyone has probably gone to bed and its quiet I would be like ‘no I’m actually struggling now’. Obviously it is the times when you’re alone which is when you tend to give in.* [IV09, pregnant, smoking]

## “Human”: Perceived Advantages of Interpersonal Support

### Accountability and Sustaining Engagement

While some felt that the anonymity offered by a digital package could be beneficial, for others interpersonal support was accompanied by an enhanced sense of accountability and they questioned the degree to which digital support could replicate this. Sustaining engagement with digital compared to human support was thought by some to be more challenging. Although receptive to the concept, some participants doubted their ability to persist in engaging with an app, citing experience of downloading apps with good intentions, but that enthusiasm can wane and “to just not bother with it” [IV12 pregnant, smoking]. It was thought that interpersonal support could be less easy to disengage from:

“*Having somebody there to say ‘Right this is what you’re going to do, this is the process you’re going to take, and we’re here to sort of support you’ - that’s like ‘Okay right I’ve got to do it’ because there’s somebody else taking their time to support me and help me…saying to you ‘Right have you met that goal?’”* [IVO5 pregnant, abstinent]

### Relationship-Based Support

Some participants who had accessed interpersonal support highlighted the value they placed on a trusted relationship and doubted that the rapport and empathy could be fully transferrable to digital: **“**That sort of empathy on the other end if you know what I mean…sometimes you do just need that person experience.” [IV13 pregnant, abstinent].

### Holistic Support

*S*ome participants also spoke about the value of human insight as part of interpersonal support, to enable holistic understanding and personalized delivery of support which addresses various aspects of an individual’s life/ social context to help facilitate smoking cessation. For example, one participant described life challenges causing a quit attempt to falter and receiving support attuned to this: “[My] midwife referred me to a different service as well, it’s like by cutting down my stress levels means that my smoking is reduced.” [IV16 pregnant, smoking].

### Reaching the Digitally Excluded

Although this did not feature prominently, digital exclusion was raised by some participants. Having sufficient storage space to download additional apps was discussed, as was affordability of paid “upgrades.”

## Engagement Enhancers

Supplementary Material 3 provides a summary of views on engagement enhancers for digital support alongside illustrative quotes. Selected key features are presented in Table 2 and discussed below.

**Table 2.** Participant Views on Engagement Enhancers for Digital Support^*^

Component	Perceived advantages	Perceived disadvantages
**Providing pregnancy-specific information**	• Enhances engagement: Positive appeal of pregnancy-related content • Inclusion of postpartum information eg, around breastfeeding	• Duplication of information already provided by pregnancy apps • Individual preference to have apps clearly delineated by purpose
**Badges and rewards** eg, for quitting progress or engagement	• Acknowledgement of progress—taking pride in achievement, earning virtual rewards • Fosters social comparison, competitiveness, engaging partners, and significant others • Utilization of small tangible rewards may still be incentivizing	• A patronizing tone when congratulating achievements is discouraging • Virtual badges can be viewed as childish or an “empty gesture” • Potential for “fraudulent” use, if utilizing financial/voucher incentives
**Notifications and prompts**	• Facilitates engagement and action—prompt to visit the app and input information • Provides a sense of support—“feeling you are not alone” • Convenience and immediacy when compared to standard interpersonal support • Can be tailored to the individual—timing and frequency of notifications, messages that reflect individual quit methods and journey	• Frequent notifications can be overwhelming and/or irritating • Concerns about privacy: visibility of notifications and disclosure of pregnancy or smoking status • Potential for waning engagement, can be easily ignored • Fear that notifications may trigger cravings
**Tracking progress**	• Highly motivational and helps foster a sense of achievement • Tracking financial savings is incentivizing, particularly when planning for a new baby • Regularity and continuity of monitoring and feedback • Ability to view visual representation of progress • Autonomy—a sense of control over quit attempt	• Doubts about sustaining usage, usefulness may be temporary • Requires time and effort (in context of busy lives) • Requires honest appraisal and input of smoking behavior
**Online forum**	• Share experience with others who understand • Ability to be honest, less fear of being judged • Ability to share experience anonymously	• Potential for misinformation and/or negative interaction, so will likely require moderation • Hesitance to actively post • Forums with little active engagement are off-putting
**Automated chatbot**	• Utility as a “first port of call”: potential to integrate escalation to “real person” • Accessibility and immediacy of feedback • Anonymity can be preferable to real person interaction	• Can be frustrating if unable to deal with user requests or needs, especially for more complex situations • Lack human contact or understanding
**Access to an advisor I**nstant messaging, voice, or video call	• Human contact and understanding • Comfort and reassurance from knowledge that a real person is available (potentially 24/7) • Human accountability and empathy	• Lack of relationship-based support or consistency (different advisers)
**Remote carbon monoxide monitoring**	• More accessible and convenient having a personal monitor than needing to visit clinic • Reinforcement and validation of achievement • Sense of autonomy and self-management • Immediacy of feedback • Appeal of a gadget • Increasing knowledge of impact of smoking through visible indication of changing CO levels • Engaging partners and significant others by sharing tools	• Potential to “cheat”—deliberately take false readings • Potential for inaccurate readings or technical problems
**Remote provision for nicotine substitution:** Provision of free nicotine replacement therapy (NRT), and potentially vapes, without interpersonal support	• More accessible and convenient to digitally order products and have home delivery • Digital modes could help facilitate NRT adherence • Potential utility to address barriers to uptake (eg, reducing stigma around approaching a pharmacist or healthcare professional) • Role for digital support to address misconceptions around e-cigarette use and safety by providing consistent information	• Potential manipulation of the system if there is no identity check • Preference to have a trained advisor overseeing use and for trouble shooting • Requires reassurance that the source of advice and delivery is reputable

^*^See Supplementary Material 3 for an expanded version with illustrative quotes drawn from interview data.

### Tracking Progress

Tracking features, for example the number of smoke-free days or money saved, were viewed as helpful to sustaining engagement, particularly as financial planning in preparation for their baby was pertinent. Those with experience of smoking cessation apps tended to report this as the feature most actively used and enjoyed due to the visual representation of progress and continuity of monitoring and feedback. Some suggested that they would benefit from pregnancy-specific feedback, outlining health benefits to their baby and integration of information on pregnancy progress as they enjoyed pregnancy-related content and suggested that this might “*bring some positivity back into it”* [IV05 pregnant, abstinent]. Nonetheless, doubts about sustaining engagement were also expressed: “I’ll be honest, I think I would forget to do it.” [IV17 pregnant, smoking].

### Remote Carbon Monoxide (CO) Monitoring

Interviewees were enthusiastic about the idea of remote CO monitoring via a specialist device and linked app. Some spoke from direct experience of this as part of a local stop smoking service initiative. The immediacy of feedback, sense of autonomy, enhanced accountability, validation of achievement, increasing knowledge of the health impact of smoking, potential to link to tailored support, alongside the appeal of a gadget were all cited as advantages: “It’s something you can do and physically see… you’ve got more of an investment in it.” [IV17 pregnant, smoking]. Household access to a personal device was also seen as having utility for engaging partners and significant others. However, reservations were expressed about the potential to “cheat” by falsifying readings.

### Notifications

A role for notifications to help avert the risk of lapse was suggested, although some feared these could be counterproductive and trigger cravings. This was also seen as offering immediacy compared with the intermittent frequency of “checking in” via interpersonal support. Preferences were expressed for notification timing to coincide with receptivity and messages tailored to individual quit methods and progress. There were some concerns about the visibility of notifications on a screen, resulting in unwanted disclosure of their pregnancy or smoking status to others.

### Rewarding Engagement

The perceived advantages of virtual rewards for progress or engagement with the digital resource included enhancing motivation and acknowledging progress. Some, however, disliked the patronizing tone or perception of virtual badges as an ‘empty gesture’ and suggested different kinds of rewards that were “more useful than a badge” [IV20 pregnant, smoking], even if these were minimal. Suggestions included “money off” or shopping vouchers, items for the baby or a posted certificate, although this raised the possibility of “fraudulent” use if material incentives or rewards were incorporated.

### Online Support Groups

An online support group as a feature of digital support was viewed positively, enabling users to seek advice from “someone that’s been in the same boat” [IV12 pregnant, smoking] with anonymity and without judgement. This was compared to some experiences with HCPs, perceived as being less able to relate. However, the potential for negative interaction, misinformation and the need for moderation was also highlighted. Some felt they would be hesitant to post, while at the same time acknowledging that forums with little current or active engagement are off-putting.

### Virtual Advisor

A “chatbot” facility was felt to have utility for those preferring more anonymous interaction, as one participant put it: “A chatbot won’t judge you.” [IV23 pregnant, abstinent]. It was also seen as offering convenient immediacy of feedback, compared to real person advice. The incorporation of an option to escalate to an interpersonal advisor, when answers to questions were not adequate, was suggested. Trouble-shooting scenarios such as CO monitor issues or NRT advice were seen as better suited to the format than more complex behavioral support, a chatbot being seen as frustrating if it does not satisfactorily address complex situations.

### Interpersonal Advice

The idea of access to interpersonal advice as a feature of a digital support package, such as asynchronous ‘chat’ communication, was welcomed: “digital support] is brilliant and you’re sort of in control of everything, but I think you need a bit of support from a healthcare professional as well.” [IV23 pregnant, abstinent]. The possibility of out-of-hours availability was particularly appealing.

### Remote Provision for Nicotine Substitution

Many participants were comfortable with the idea of remote provision for nicotine substitution (NRT and e-cigarettes) without interpersonal support. They felt that digital support from a trusted provider (eg, NHS) could facilitate access to and use of NRT during pregnancy, especially for those who were already familiar with the products: “…you don’t need to go through someone to order… you have control.” [IV23 pregnant, abstinent]. Automated notifications to enhance NRT adherence, such as a reminder to apply a patch, were also suggested. Some felt that they would be less confident without the support of an advisor, suggesting an initial interpersonal advice component before going on to utilize a digital service. Remote digital provision was, however, viewed as removing the hassle of repeat ordering products, both in terms of convenience and speed of receipt:

*“Whenever I needed new stuff, I had to get in touch with the advisor to speak to the pharmacy for me to then go and pick it up so there’s a lot of waiting around or if I couldn’t get hold of her I’d kind of worry am I going to hear from her in time for any patches or whatever...if it was being delivered...I wouldn’t have to really worry about it.”* [IV10 postpartum, smoking]

There was limited awareness among some participants about the range of NRT or e-cigarette products available, and some who had previous unsuccessful experience with NRT or vaping felt that encouragement to revisit this may be helpful, for example, by sampling alternative products or trying different doses. Some concerns were raised about the potential for manipulation of the system if there is no identity check.

A minority of participants who had used or considered using e-cigarettes to support their quit experienced conflicting advice or misinformation. It was suggested that a reputable digital support package may help to address this, by incorporating myth-busting information and providing support on switching to vaping.

## Initiation, Implementation and Integration

Participants were keen to receive cessation support at an early point in pregnancy, prior to their ‘booking appointment’ before 10 weeks. There had been no option for this, as one participant put it: “…four weeks I had to wait before I then met my midwife - had I had support then maybe I might have quit a bit sooner” [IV07 pregnant, abstinent]. Participants viewed early access to a digital support package as an important opportunity for a “head start*”* [IV11 pregnant, smoking].

Participants raised the importance of an external prompt to action to use digital support. Signposting opportunities suggested by participants in addition to an HCP included linkage to maternity self-referral forms and Electronic Patient Record portals/app, pregnancy app signposting, social media advertising or via early contact opportunities such as GP or Early Pregnancy Unit appointments.

Three clear pathways were expressed by participants for how a digital support package could work: (1) Stand-alone support independent of and in preference to in-person support, (2) Parallel adjunct support alongside NHS interpersonal cessation support, providing enhanced behavioral support and ongoing motivation and support in-between appointments, (3) Parallel integrated support whereby data from the digital support is shared with an advisor or HCP (eg, days smoke-free or CO readings).

Finally, participants felt a pregnancy-specific digital package should provide support beyond birth, to prevent relapse. Some participants identified this as a support gap and a wasted opportunity: “what’s the point of giving up smoking during pregnancy if once the baby is here you’re going to start smoking again.” [IV07 pregnant, abstinent].

## Discussion

This study provides new insights into views on a digital support package for smoking cessation during pregnancy. Many participants were enthusiastic about a digital alternative to standard interpersonal treatment. It was thought that pregnancy-specific digital support, particularly a smartphone app, would be more convenient and nonjudgmental, provide better consistency of advice, and enhance privacy and autonomy. Digital support was particularly appealing to those juggling family and work commitments or feeling anxious; with one in five pregnant women in high-income countries experiencing antenatal anxiety.^33^ Digital interventions may be particularly suited to those who are reluctant to disclose health problems because of stigma or fear this might impact on the support they receive from HCPs.^34^

Despite identifying various advantages to digitalizing stop smoking support in pregnancy, some participants identified limitations, especially that, in contrast, interpersonal support can help to foster a sense of accountability, empathy, and address the wider life challenges that impact on smoking behavior. It was apparent that while a digital support package might be initially appealing, potentially improving uptake of cessation support in pregnancy, sustaining engagement may be challenging. The issue of engagement appears to be more apparent for apps, including for a prototype smoking in pregnancy app that was terminated due to low engagement,^19^ than for text message support.^35^ This may be because app notifications are more easily muted, switched off or ignored and most app features require proactive engagement, compared with text messages. Digital literacy has also been found to be associated with app use.^36^ A future cessation intervention for pregnancy may benefit from including a combination of delivery modes.

In this study, various engagement enhancers for digital support were identified, including the ability to track progress through digital recording tools and the use of remote CO monitoring. While the former is a frequently used behavior change technique in generic cessation apps and a preferred user feature, its efficacy is uncertain.^37,38^ Remote CO monitoring is a relatively new technology that allows individuals to track their CO levels using a personal device linked to their smartphone. Service evaluations indicate that this may be motivational for pregnant service users,^39^ but cost-effectiveness is uncertain.

Other approaches to increase engagement with digital support included retaining some human involvement; by being able to communicate with a cessation specialist if needed and providing online social support. This can improve outcomes,^40–42^ but the involvement of cessation specialists also increases costs. Peer social support is a potentially low-cost strategy to increase engagement and appealing to pregnant smokers. However, evidence suggests the effects are unclear.^4^ Social media cessation interventions in the general populations have shown promise but are dependent on participant engagement^39^ and might require moderation to avoid the spread of misinformation. Offering digital support to partners and significant others could be a further way to boost social support and is highlighted in NICE current treatment guidance.^6^

Remote provision of NRT alongside a digital support package may also improve engagement and enhance support effectiveness. Evidence from nine pooled studies shows that NRT combined with interpersonal counseling may increase the likelihood of smoking cessation in later pregnancy (RR 1.37, 95% CI: 1.08 to 1.74).^43^ Participants were generally comfortable about the idea of obtaining nicotine substitution products without interpersonal support, and digital ordering and home provision might help to overcome practical barriers to adherence. However, some participants reported having unsuccessful experiences with NRT or vaping, which they might be reluctant to try again without human support. One suggestion was to provide samples, as currently being tested in the Baby Breathe postpartum relapse trial.^44^

**Figure 1.**
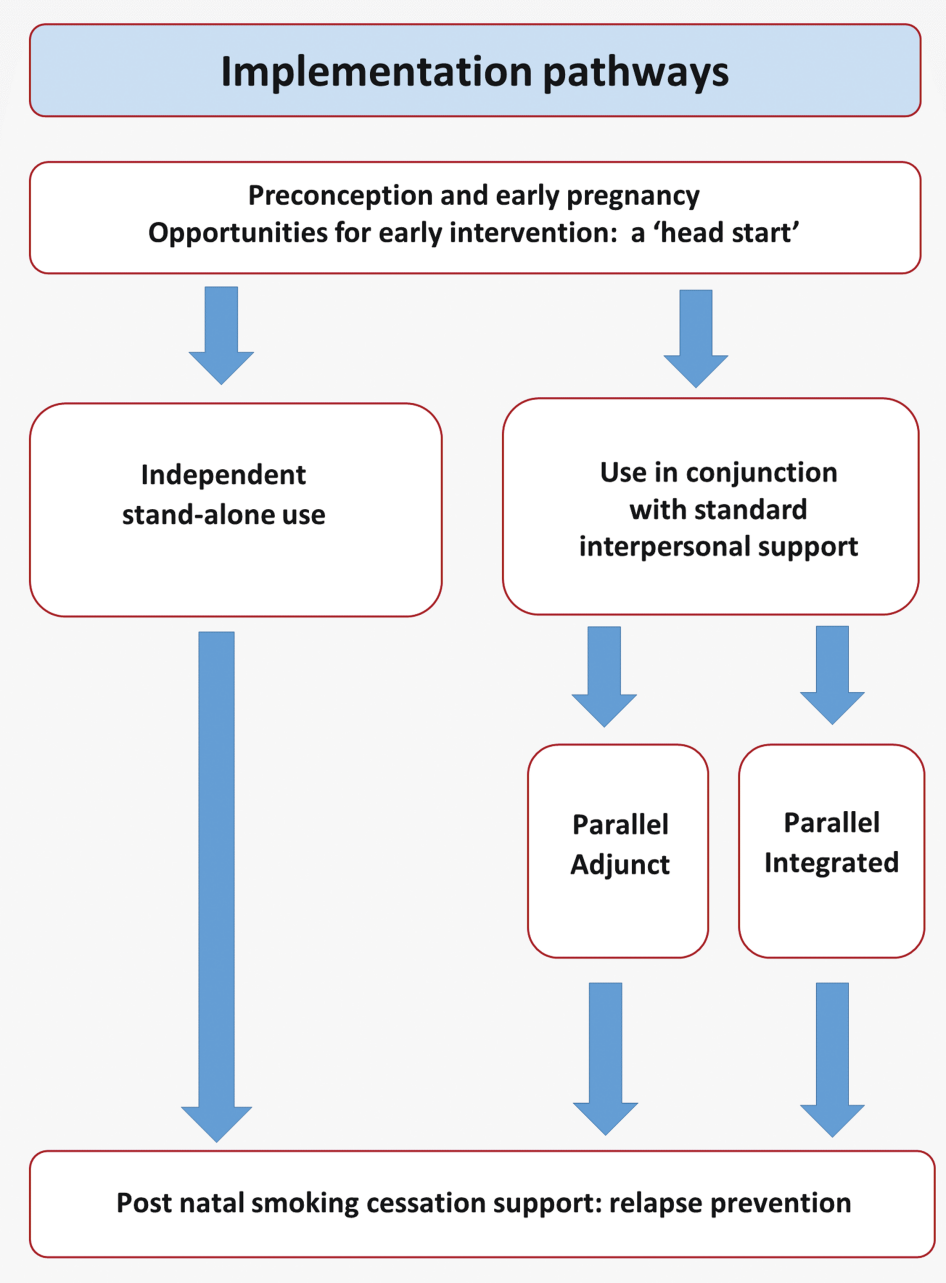
Pathways for implementation.

For our interviewees, those who engaged with the STP interpersonal support had a similar level of interest in digital support as those who did not. This also applied to quit status, with both those who had successfully or unsuccessfully quit showing similar levels of interest. Moreover, when asked hypothetically about choosing between modes of support, participants largely expressed a preference for digital modes. This suggests utility of digital support for both those who feel able to engage with STP interpersonal support and those who don’t. Not all those interviewed who had accessed STP had been able to successfully quit, citing perceived stigma, interaction preferences, inflexibility, inconvenience or lack of continuity of access as barriers. The implications are that digital support could potentially both extend the reach of smoking cessations programs by reaching those who would not otherwise engage and also provide supplementary support or a cost-effective alternative to the STP. These implications were also raised in our parallel work with smoking cessation experts,^30^ who also cautioned that, for the latter scenario, a digital intervention could inadvertently contribute towards disengagement with services if viewed as an opportunity to ‘opt out’ of interpersonal support. An important theme throughout interviews was the optimal timing for introducing digital stop smoking support in pregnancy and how this might work alongside routine maternity-led provision. An illustration of potential pathways for implementation, drawing on interview data, is presented in Figure 1. The need for digital support to come from a credible source was identified, and evidence shows this is important for initiation.^34^ Digital support was seen to work alongside routine care in various ways. Firstly, as a stand-alone intervention, helping to widen access for those who might not want to quit with “human-assisted” support. Secondly, some participants said they would want to access digital support alongside standard treatment, and finally, for the digital data collected to be shared with HCPs on an optional basis to help enhance the support they offer. Investigating whether these different combinations may increase the overall numbers successfully quitting smoking in pregnancy is a task for future research.

Various initiation points were identified for digital support with the advantage that this could facilitate timely access when people need it most; in particular, digital support may have a role in early pregnancy, which is important as quitting smoking in the first trimester provides the greatest benefits.^45^

## Strengths and Limitations

A key strength of this study is the diversity of the sample in terms of terms of age, ethnicity, geographical location, smoking status, and degree of access to stop smoking support. We strived to reduce barriers to participation in our recruitment approach (eg, inclusive imagery, and language) and in the conduct of the study (eg, flexible interview timing and formats). However, the inclusion criterion stipulating that participants needed to have made a quit attempt in their recent pregnancy which may have resulted in a more motivated sample, and interested participants were aware of the study focus so might have been more open to digital support. A further limitation is reliance on perceptions of interest and use. This can be overcome incorporating theory and evidence of engagement enhancers into intervention design^46^ and using think-aloud methods to^47^ to help identify preferred intervention features.

## Conclusions

Digital smoking cessation support for pregnancy was attractive to our sample and was felt to overcome many barriers to engaging with interpersonal support. However, some participants felt that fully removing access to a human could undermine a digital intervention and reduce engagement. The findings support the investigation of digital support as both a stand-alone package and adjunct to standard interpersonal cessation support in pregnancy to increase the proportion of pregnant smokers who make a supported quit attempt.

## Supplementary Material

Supplementary material is available at *Nicotine and Tobacco Research* online.

ntae184_suppl_Supplementary_Material_S1

ntae184_suppl_Supplementary_Material_S2

ntae184_suppl_Supplementary_Material_S3

## Data Availability

The anonymized data that support the findings of this study are available on request from the corresponding author, PB. The data are not publicly available due to ethics restrictions.

## References

[1] Salihu HM , Wilson RE. Epidemiology of prenatal smoking and perinatal outcomes. Early Hum Dev. 2007;83(11):713–720. doi: https://doi.org/10.1016/j.earlhumdev.2007.08.002 17884310

[2] Avşar TS , McLeod H, Jackson L. Health outcomes of smoking during pregnancy and the postpartum period: an umbrella review. BMC Pregnancy Childbirth. 2021;21(1):254. doi: https://doi.org/10.1186/s12884-021-03729-1 33771100 PMC7995767

[3] Public Health England. Characteristics of women who stop smoking in pregnancy: Experimental analysis of smoking data from the Maternity Services Data Set (MSDS), April 2018 to March 2019. 2020. https://www.publishing.service.gov.uk. Accessed July 29, 2024.

[4] Chamberlain C , O’Mara‐Eves A, Porter J, et al Psychosocial interventions for supporting women to stop smoking in pregnancy. Cochrane Database Syst Rev. 2017;2(2):CD001055. doi: https://doi.org/10.1002/14651858.CD001055.pub5 28196405 PMC6472671

[5] Bar‐Zeev Y , Lim LL, Bonevski B, Gruppetta M, Gould GS. Nicotine replacement therapy for smoking cessation during pregnancy. Med J Aust. 2018;208(1):46–51. doi: https://doi.org/10.5694/mja17.00446 29320660

[6] National Institute for Health and Care Excellence (NICE). NICE guideline [NG209]: Tobacco: preventing uptake, promoting quitting and treating dependence. Recommendations on treating tobacco dependence in pregnant women; 2021. https://www.nice.org.uk/guidance/ng209/chapter/Recommendations-on-treating-tobacco-dependence-in-pregnant-women. Accessed 30 July, 2023. 38598649

[7] National Centre for Smoking Cessation and Training (NCSCT). Standard treatment programme for pregnant women: a guide to providing behavioural support for smoking cessation during pregnancy and the post-partum period; 2019. https://www.ncsct.co.uk/library/view/pdf/NCSCT%20Standard%20Treatment%20Programme%20for%20Pregnant%20Women.pdf

[8] National Institute for Health and Care Excellence. PH26 effectiveness of interventions during pregnancy: briefing paper. 2021. https://www.nice.org.uk/guidance/ng209/evidence/ph26-effectiveness-of-interventions-during-pregnancy-briefing-paper-pdf-10890920418

[9] Naughton F , Vaz LR, Coleman T, et al Interest in and use of smoking cessation support across pregnancy and postpartum. Nicotine Tob Res. 2020;22(7):1178–1186. doi: https://doi.org/10.1093/ntr/ntz151 31570944 PMC7291796

[10] Campbell KA , Cooper S, Fahy SJ, et al ‘Opt-out’referrals after identifying pregnant smokers using exhaled air carbon monoxide: impact on engagement with smoking cessation support. Tob Control. 2017;26(3):300–306. doi: https://doi.org/10.1136/tobaccocontrol-2015-052662 27225017 PMC5520259

[11] Thomson R , Cooper S, Waldron J, et al Smoking cessation support for pregnant women provided by english stop smoking services and national health service trusts: a survey. Int J Environ Res Public Health. 2022;19(3):1634. doi: https://doi.org/10.3390/ijerph19031634 35162656 PMC8835166

[12] Griffiths SE , Naughton F, Brown KE. Accessing specialist support to stop smoking in pregnancy: a qualitative study exploring engagement with UK‐based stop smoking services. Br J Health Psychol. 2022;27(3):802–821. doi: https://doi.org/10.1111/bjhp.12574 34852182 PMC9542141

[13] Ussher M , Etter J-F, West R. Perceived barriers to and benefits of attending a stop smoking course during pregnancy. Patient Educ Couns. 2006;61(3):467–472. doi: https://doi.org/10.1016/j.pec.2005.06.021 16098707

[14] Thomson R , Cooper S, Waldron J, et al Smoking cessation support for pregnant women provided by English stop smoking services and National Health Service Trusts: a survey. Int J Environ Res Public Health. 2022;19(3):1634. doi: https://doi.org/10.3390/ijerph19031634 35162656 PMC8835166

[15] National Health Service (NHS). The NHS Long Term Plan. 2019. https://www.longtermplan.nhs.uk/wp-content/uploads/2019/08/nhs-long-term-plan-version-1.2.pdf. Accessed July 31, 2023.

[16] Grant A , Morgan M, Gallagher D, Mannay D. Smoking during pregnancy, stigma and secrets: visual methods exploration in the UK. Women Birth. 2020;33(1):70–76. doi: https://doi.org/10.1016/j.wombi.2018.11.012 30553588 PMC7043392

[17] Butterworth SJ , Sparkes E, Trout A, Brown K. Pregnant smokers’ perceptions of specialist smoking cessation services. J Smok Cessat. 2014;9(2):85–97. doi: https://doi.org/10.1017/jsc.2013.25

[18] Cooper S , Orton S, Leonardi-Bee J, et al Smoking and quit attempts during pregnancy and postpartum: a longitudinal UK cohort. BMJ Open. 2017;7(11):e018746. doi: https://doi.org/10.1136/bmjopen-2017-018746 PMC5695489 29146659

[19] Tombor I , Beard E, Brown J, et al Randomized factorial experiment of components of the SmokeFree Baby smartphone application to aid smoking cessation in pregnancy. Transl. Behav. Med.. 2019;9(4):583–593. doi: https://doi.org/10.1093/tbm/iby073 30011020 PMC6629841

[20] Coleman T , Clark M, Welch C, et al Effectiveness of offering tailored text message, self‐help smoking cessation support to pregnant women who want information on stopping smoking: MiQuit3 randomised controlled trial and meta‐analysis. Addiction. 2022;117(4):1079–1094. doi: https://doi.org/10.1111/add.15715 34636086

[21] Herbec A , Brown J, Tombor I, Michie S, West R. Pilot randomized controlled trial of an internet-based smoking cessation intervention for pregnant smokers (“MumsQuit”). Drug Alcohol Depend. 2014;140(1):130–136. doi: https://doi.org/10.1016/j.drugalcdep.2014.04.010 24811202 PMC4067748

[22] Abroms LC , Johnson PR, Leavitt LE, et al A randomized trial of text messaging for smoking cessation in pregnant women. Am J Prev Med. 2017;53(6):781–790. doi: https://doi.org/10.1016/j.amepre.2017.08.002 28982527 PMC5696101

[23] Griffiths SE , Parsons J, Naughton F, et al Are digital interventions for smoking cessation in pregnancy effective? A systematic review and meta-analysis. Health Psychology Review. 2018;12(4):333–356. doi: https://doi.org/10.1080/17437199.2018.1488602 29912621

[24] Marin-Gomez FX , Marchán RG-M, Mayos-Fernandez A, et al Exploring efficacy of a serious game (Tobbstop) for smoking cessation during pregnancy: randomized controlled trial. JMIR Serious Games. 2019;7(1):e12835. doi: https://doi.org/10.2196/12835 30916655 PMC6456830

[25] Riaz M , Lewis S, Naughton F, Ussher M. Predictors of smoking cessation during pregnancy: a systematic review and meta‐analysis. Addiction. 2018;113(4):610–622. doi: https://doi.org/10.1111/add.14135 29235189

[26] Emery JL , Sutton S, Naughton F. Cognitive and behavioral predictors of quit attempts and biochemically-validated abstinence during pregnancy. Nicotine Tob Res. 2017;19(5):547–554. doi: https://doi.org/10.1093/ntr/ntw242 28403458 PMC5896485

[27] Herbec A , Beard E, Brown J, et al The needs and preferences of pregnant smokers regarding tailored Internet-based smoking cessation interventions: a qualitative interview study. BMC Public Health. 2014;14(1):1070. doi: https://doi.org/10.1186/1471-2458-14-1070 25312556 PMC4209063

[28] Tong A , Sainsbury P, Craig J. Consolidated criteria for reporting qualitative research (COREQ): a 32-item checklist for interviews and focus groups. Int J Qual Health Care. 2007;19(6):349–357. doi: https://doi.org/10.1093/intqhc/mzm042 17872937

[29] Braun V , Clarke V. To saturate or not to saturate? Questioning data saturation as a useful concept for thematic analysis and sample-size rationales. Qual Res Sport Exerc Health. 2021;13(2):201–216. doi: https://doi.org/10.1080/2159676X.2019.1704846

[30] McDaid L , Belderson P, Emery J, et al Experts’ views on translating NHS support to stop smoking in pregnancy into a comprehensive digital intervention. PLOS Digit Health. 2024;3(3):e0000472. doi: https://doi.org/10.1371/journal.pdig.0000472 38536890 PMC10971751

[31] Braun V , Clarke V. Using thematic analysis in psychology. Qual Res Psychol. 2006;3(2):77–101. doi: https://doi.org/10.1191/1478088706qp063oa

[32] Office of National Statistics (ONS). ONS Postcode Directory. 2023. https://geoportal.statistics.gov.uk/datasets/a2f8c9c5778a452bbf640d98c166657c/about. Accessed June 6, 2023.

[33] Dennis C-L , Falah-Hassani K, Shiri R. Prevalence of antenatal and postnatal anxiety: systematic review and meta-analysis. Br J Psychiatry. 2017;210(5):315–323. doi: https://doi.org/10.1192/bjp.bp.116.187179 28302701

[34] Evans K , Rennick-Egglestone S, Cox S, Kuipers Y, Spiby H. Remotely delivered interventions to support women with symptoms of anxiety in pregnancy: mixed methods systematic review and meta-analysis. J Med Internet Res. 2022;24(2):e28093. doi: https://doi.org/10.2196/28093 35166688 PMC8889484

[35] Naughton F , Cooper S, Foster K, et al Large multi‐centre pilot randomized controlled trial testing a low‐cost, tailored, self‐help smoking cessation text message intervention for pregnant smokers (MiQuit). Addiction. 2017;112(7):1238–1249. doi: https://doi.org/10.1111/add.13802 28239919 PMC5488183

[36] Szinay D , Jones A, Chadborn T, Brown J, Naughton F. Influences on the uptake of and engagement with health and well-being smartphone apps: systematic review. J Med Internet Res. 2020;22(5):e17572. doi: https://doi.org/10.2196/17572 32348255 PMC7293059

[37] Zhang M , Wolters M, O’connor S, Wang Y, Doi L. Smokers’ user experience of smoking cessation apps: a systematic review. Int J Med Inform. 2023;178:105069. doi: https://doi.org/10.1016/j.ijmedinf.2023.105069 37084673

[38] Bize R , Burnand B, Mueller Y, et al Biomedical risk assessment as an aid for smoking cessation. Cochrane Database Syst Rev. 4705;2012(12):CD00. doi: https://doi.org/10.1002/14651858.CD004705.pub5 23235615

[39] Hellyar T , Marais, F.. Integrating iCOquit monitors to reduce smoking during pregnancy in Somerset: a mixed-method evaluation research. 2022; Somerset Council, UK. https://ash.org.uk/uploads/iCO-Monitor-Evaluation-summary.pdf?v=1671707015. Accessed July 30, 2023.

[40] Werntz A , Amado S, Jasman M, Ervin A, Rhodes JE. Providing human support for the use of digital mental health interventions: systematic meta-review. J Med Internet Res. 2023;25(2):e42864. doi: https://doi.org/10.2196/42864 36745497 PMC9941905

[41] Webb J , Peerbux S, Smittenaar P, et al Preliminary outcomes of a digital therapeutic intervention for smoking cessation in adult smokers: randomized controlled trial. JMIR Ment Health. 2020;7(10):e22833. doi: https://doi.org/10.2196/22833 33021488 PMC7576529

[42] Naslund JA , Kim SJ, Aschbrenner KA, et al Systematic review of social media interventions for smoking cessation. Addict Behav. 2017;73:81–93. doi: https://doi.org/10.1016/j.addbeh.2017.05.002 28499259 PMC5556947

[43] Claire R , Chamberlain C, Davey MA, et al Pharmacological interventions for promoting smoking cessation during pregnancy. Cochrane Database Syst Rev. 2020;3(3):CD010078. doi: https://doi.org/10.1002/14651858.CD010078.pub3 32129504 PMC7059898

[44] Notley C , Brown TJ, Bauld L, et al Development of a complex intervention for the maintenance of postpartum smoking abstinence: process for defining evidence-based intervention. Int J Environ Res Public Health. 2019;16(11):1968. doi: https://doi.org/10.1136/bmj.b1081 31163663 PMC6603989

[45] McCowan LM , Dekker GA, Chan E, et al; SCOPE consortium. Spontaneous preterm birth and small for gestational age infants in women who stop smoking early in pregnancy: prospective cohort study. BMJ. 2009;338(7698):b1081. doi: https://doi.org/10.1136/bmj.b1081 19325177 PMC2661373

[46] Yardley L , Spring BJ, Riper H, et al Understanding and promoting effective engagement with digital behavior change interventions. Am J Prev Med. 2016;51(5):833–842. doi: https://doi.org/10.1016/j.amepre.2016.06.015 27745683

[47] Yardley L , Morrison L, Bradbury K, Muller I. The person-based approach to intervention development: application to digital health-related behavior change interventions. J Med Internet Res. 2015;17(1):e30. doi: https://doi.org/10.2196/jmir.4056 25639757 PMC4327440

