# Supplementary material for: Digitalizing Specialist Smoking Cessation Support in Pregnancy: Views of Pregnant Smokers
Source: Nicotine Tob Res. 2024 Jul 26;27(2):225–35. doi: 10.1093/ntr/ntae184 (PMC11750734; doi:10.1093/ntr/ntae184)
Supplement: ntae184_suppl_Supplementary_Material_S1 [file ntae184_suppl_supplementary_material_s1.docx]

**Supplementary Material 1**

**eSupport Study for Smoking Cessation in Pregnancy**

**Topic guide for pregnant people**

**1) Quit and eSupport experience**

1. ***Please tell me how you got on with trying to quit smoking in pregnancy?*** (Prompt: was the quit attempt(s) successfully or not)
2. ***Did you access any support to help you try stop smoking in pregnancy? If so, please tell me about your experience.*** (Prompts: type of support / how heard about it/ which parts of the standard treatment programme e.g. information, planning, carbon monoxide (CO) testing, NRT, and how delivered / how long engaged with support)
3. ***Have you used any digital support for stopping smoking in pregnancy or searched for help or information online?*** (Prompts: If yes, explore what apps or websites and features - did they get a chat bot, advisors, could they get NRT, did they get a quit plan, did the app support multiple quit attempts?)

**2) Potential features of eSupport for stopping smoking in pregnancy (think aloud approach)**

The standard NHS stop smoking treatment programme for pregnant people involves six sessions delivered by a trained advisor. We want to explore ways this can be delivered without a human advisor. ***We’d like to do this by going through the sheet we provided to talk to you about your thoughts on the different ways this support might be delivered***. You may want to think about any stop smoking support you received when you answer.

We want your honest feedback, so don’t feel you have to be positive about anything. If you don’t like the sound of any of these forms of support, then please tell me about that - we are keen to hear about this too!

**(**Prompts: How helpful? How usable?; Advantages and disadvantages**;** Situations that it might be more or less useful for; How could it be made more helpful, more usable? How long might you use it for/find it useful? Prompt comparison of forms of support.)

**3) Structuring e-support**

- ***Thinking about your use of digital tools in general, what features encourage you to go back on a daily/ regular basis?*** (Prompts: reminders, quiz questions, new information, monitoring). Do you have any other suggestions on how to keep people engaged?)
- ***Thinking about digital tools generally, I’d like to ask you about what delivery methods may work best.*** (Prompts: a structured programme where you had guided counselling sessions with a virtual stop smoking expert and tasks to complete each week, bitesize information to look at as and when you need it, option to choose the level of help you need)
- ***What do you think about the idea of partners or other smokers in the household being offered e-support?***

**4) Scenario-based *prompts*** *(NOTE: this content is flexible, depending on time and what has already been spoken about during scoresheet discussion).* For the next part of the interview, I’m going to ask you to imagine yourself in some different situations.

- ***Nicotine substitution: If you were given an app to download which let you order NRT to be delivered to your home, would you use it?*** (Prompts: would you have any concerns about ordering NRT without speaking to an advisor? What are your views about advice or access to an e-cigarette as part of the support?)
- ***Imagine you were offered the option of a digital stop smoking support package or practitioner stop smoking support, which would you choose and why?***
- ***Imagine you had successfully quit during pregnancy but after you’ve had the baby you start smoking again. You receive a notification checking in with you about whether you are smoking. How might you feel about this?***

**Initiation and implementation**

- ***If the NHS wanted to promote an eSupport package to help you stop smoking, what would be the best way to do this?*** (Prompt: health professional referral, internet adverts, being emailed/ texted about it)
- ***Thinking back to when you first informed a health service about your pregnancy – who did you first inform?*** (Prompts: how would you feel if they had suggested an e-support option for stopping smoking? What would be your thoughts on an opt-out model where, as someone who smoked, you would automatically be signed up to some ‘light touch’ digital support to stop smoking during pregnancy unless you chose to opt out of this?)
- ***What if your doctor or midwife had access to some of the data that an app might collect from you, with your permission - how would you feel about sharing your progress or data at appointment with an HP?*** (Prompt: do you have concerns about confidentiality and data protection with digital support?)

**4) Closing**

- Thinking about everything we have discussed, what would be your ‘wish list’ for an eSupport package?

- Thank the participant for sharing their views and ask them if there is anything else they would like to add?
