# Supplementary material for: Digitalizing Specialist Smoking Cessation Support in Pregnancy: Views of Pregnant Smokers
Source: Nicotine Tob Res. 2024 Jul 26;27(2):225–35. doi: 10.1093/ntr/ntae184 (PMC11750734; doi:10.1093/ntr/ntae184)
Supplement: ntae184_suppl_Supplementary_Material_S2 [file ntae184_suppl_supplementary_material_s2.docx]

**Supplementary material 2**

**Potential tools and features for an ‘eSupport’ package**

**Below are some examples of different types of digital support for quitting smoking during pregnancy.**

**Please take a look at these and think about whether they would be helpful to you. We will talk through some of these as part of the interview – and would value your honest feedback!**

| **Information (written/video/audio) e.g.**   - Why it’s important to quit smoking in pregnancy - Quit tips - Myth busting - Using NRT/ vaping in pregnancy   **Image 1: Quit for You, Quit for Two app**  **Image 2: Baby Buddy app**  **Image 3: SmokeFree Norfolk podcast** | **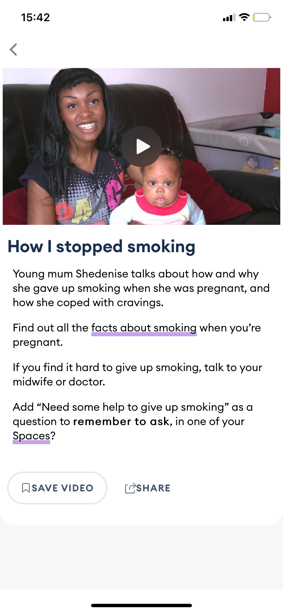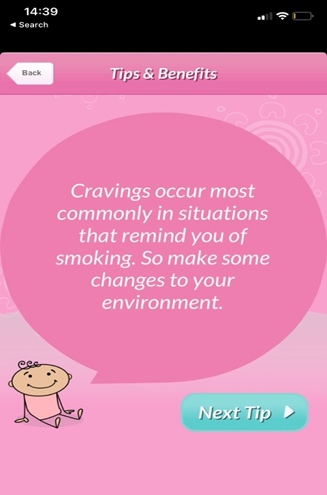**  **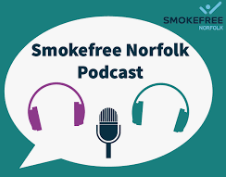** |
| --- | --- |
| **Track your progress**   - The ability to record how your quit attempt is going using a ‘daily diary’ of your smoking, use of Nicotine Replacement Therapy (NRT) and any vaping - Feedback based on your ‘daily diary’ e.g. a savings calculator or any health benefits you are getting (if not smoking)   **Image: NHS Quit Smoking app** | 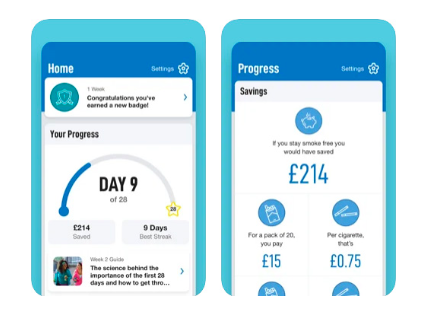 |
| **Notifications and prompts**   - Support messages coming at different times during the day to help motivate you and give you confidence in being able to quit - Tips on how to quit smoking and manage challenging situations (e.g. when stressed)   **Image 1: NHS Quit Smoking app**  **Image 2: MiQuit SMS** | 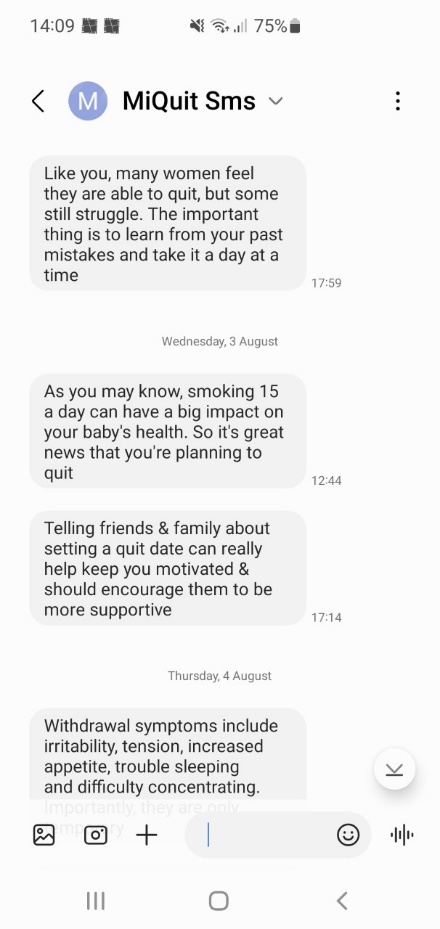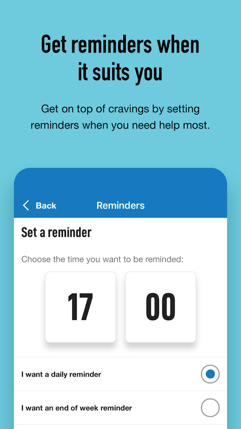 |
| **Missions / Check-ins**   - Daily missions or tasks, to motivate or help you resist cravings - Regular check-ins tailored to your quit plan, e.g. on quit date, smoking trigger points, using NRT   **Image : SmokeFree app** | 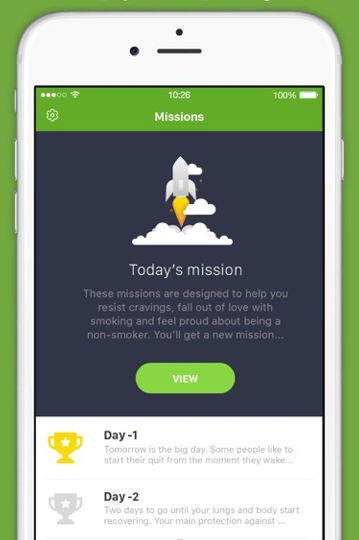 |
| **Badges or reward scheme**  Virtual rewards or badges for:   - quitting progress - engaging with different parts of the app/ website   **Image: NHS Stop Smoking app** | 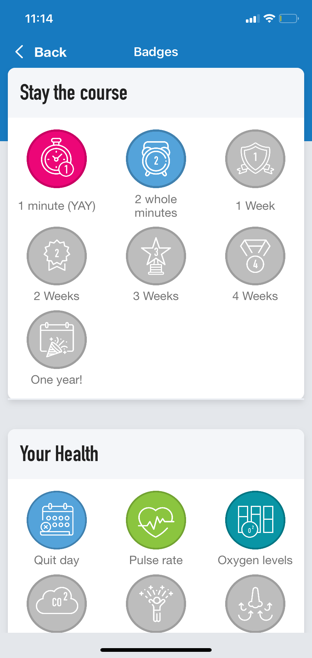 |
| **Carbon monoxide (CO) monitoring***   - Information about CO in pregnancy - Personal Bluetooth CO monitor to enable you to take CO readings, view the results on an app/online and share the results and progress with others   ******Carbon monoxide (CO) is a toxic gas found in tobacco smoke. Measuring exhaled CO with a monitor can help motivate and chart the progress of smokers trying to quit.*  **Image: iCOquit®** | **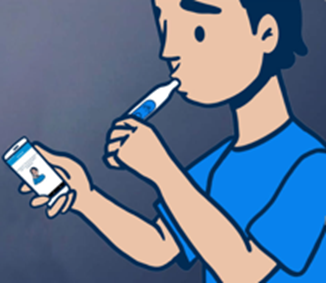** |
| **Tailoring to your personal situation**  Questions about you, your smoking routines, circumstances, preferences and progress to make content more relevant to your personal situation  **Image: NHS Stop Smoking app** | 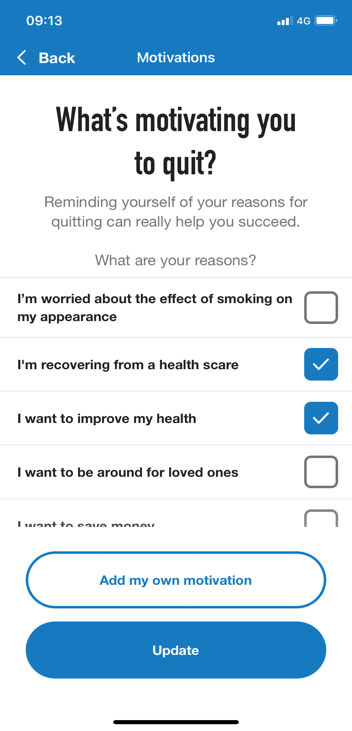 |
| **Automated Chat bot**   - Have a conversation when you need it - Programmed with automated responses such as practical advice and tips   **Image: Smoke Free app** | **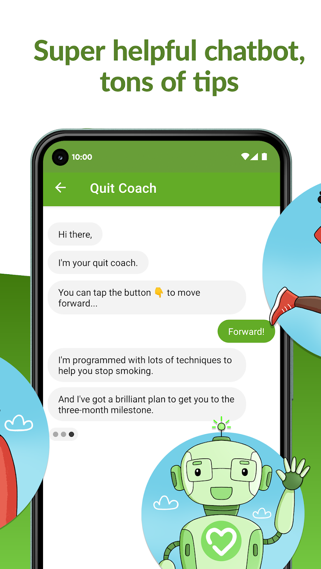** |
| **Access to an advisor (instant messaging, voice or video call)**   - Access to trained stop smoking advisors at times convenient to you. - Can answer questions and keep you motivated   **Image: Smoke Free app** | 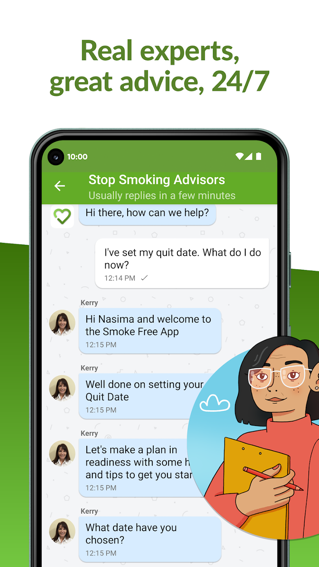 |
| **Chat with other quitters (online forum)**   - Know that you are not alone and there are others going through similar journeys - Share experiences and helpful tips with others   **Image: NHS Smokefree Facebook group** | 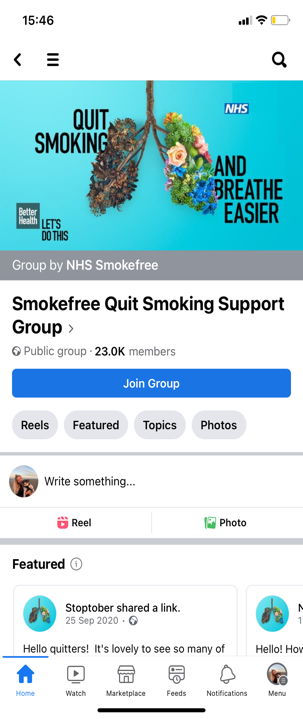 |
| **Pregnancy specific information**   - See how your pregnancy is progressing   **Image: Quit for You, Quit for Two app** | 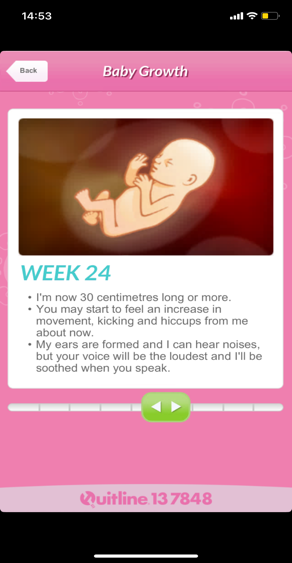 |
| **Games**   - Games to help distract you and resist cravings   **Image: NHS Stop Smoking app** | 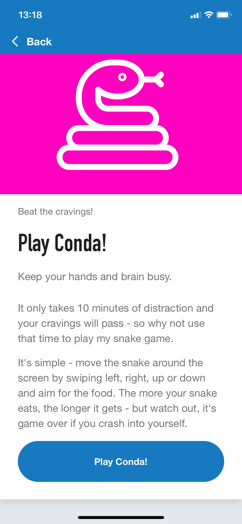 |
| **Welcome box**  Delivered through the letter box, containing for eg:   - CO monitor - Nicotine gum - Information leaflets - Progress chart   **Image: BabyBreathe** | 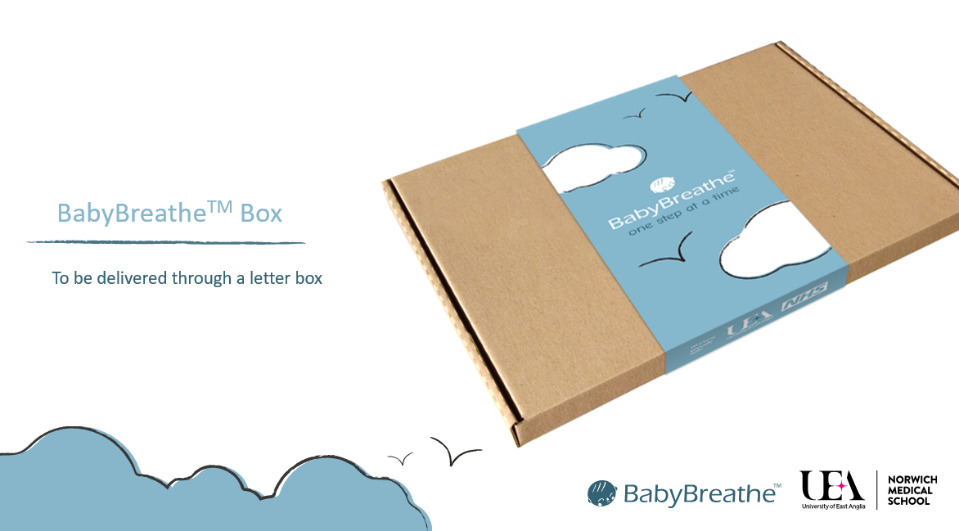 |

**Thank you for helping this research!**

**We would like to talk through your thoughts as part of the interview so please have the completed sheet to hand on the day.**

**Example resources**

If you would like to have a closer look at some example resources or have a play with some apps, we have provided links and QR codes on the this page. **This is optional** – you do not have to look at these, or might just want to pick one or two.

|  |  |  | **Videos:** |  |
| --- | --- | --- | --- | --- |
| **App:** My Quit Route | 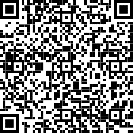 |  | - Mythbusting: 10 myths about quitting smoking in pregnancy - YouTube | **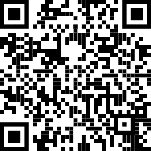** |
| **App:** Smoke Free | **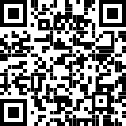** |  | - Animation: Stop Smoking in Pregnancy - YouTube | **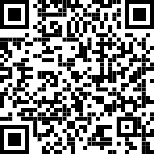** |
| **Text message support:** MiQuit SMS | **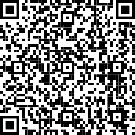** |  | - Product demonstration video**:** How to use NRT | **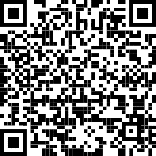** |
| **WhatsApp** WHO 14 day quit challenge | **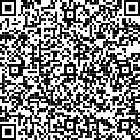** |  | **Podcast:** Smoking In Pregnancy | **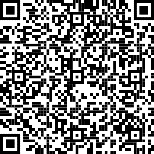** |
| **Remote CO monitoring**  How to use the iCOquit® Smokerlyzer - Video | **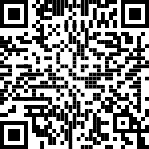** |  | **Infographic:** e-cigarettes in pregnancy | **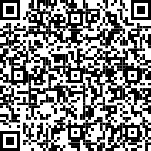** |
| **Website** Quit smoking - Better Heath (NHS) | **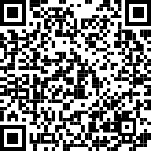** |  |  |  |

*A quick disclaimer: We cannot verify that the information is accurate or that these resources are effective in helping people quit smoking.*
