# Supplementary material for: Digitalizing Specialist Smoking Cessation Support in Pregnancy: Views of Pregnant Smokers
Source: Nicotine Tob Res. 2024 Jul 26;27(2):225–35. doi: 10.1093/ntr/ntae184 (PMC11750734; doi:10.1093/ntr/ntae184)
Supplement: ntae184_suppl_Supplementary_Material_S3 [file ntae184_suppl_supplementary_material_s3.docx]

**Supplementary material 3:  Participant views on engagement enhancers for digital support**

| **Perceived advantages and disadvantages** | | Exemplar quotes |
| --- | --- | --- |
| **Providing information** | | |
|  | **Advantages**   - A trusted digital support package as a central ‘go to’ point for consistent and reliable information - Modes of delivery to suit individual preference (written/audio/visual) - Convenience:  ability to fit in around family / work life - The ability to revisit information, for example by re-watching videos - A role for provision of ‘hard-hitting’ information - visual medium and imagery more impactful. - Ability to share information with partners and significant others, for example by sending a link. | *“I definitely think it helps to have an app that you can go on that again you know isn’t just Google where you are going to search and get a million different answers”* [IV05 pregnant, abstinent]    *“It is a lot to take in in one phone call whereas a video is something you can go back to and re-watch if you need to”* [IV22 postpartum, smoking]    *“I’ve been judged like with other professionals…I think when you look at it like on an app and like a video it’s different, you can take it sort of like at your own time, your own pace”* [IV23 pregnant, abstinent]] |
|  | **Disadvantages**   - Information alone can be perceived as repetitive. If content is not engaging or varied it may not sustain interest | *“Once it doesn’t capture your attention anymore it doesn’t, it’s not an attractive thing…then you get bored so it needs to be something that’s ongoing with new information all of the time, new ideas, new strategies”* [IV17 pregnant, smoking] |
| **Providing pregnancy specific information (how pregnancy is progressing)** | | |
|  | **Advantages**   - Enhances engagement: positive appeal of pregnancy related content. - Inclusion of post-partum information e.g. around breastfeeding | *“I think it would keep people going to the app because I know particularly with my first pregnancy I was constantly checking what size my baby was compared to a watermelon and all that sort of stuff, so if you had a smoking app that had all of those features it would probably encourage people to go on”* [IV13]    *“I have a Baby App on my phone and I go on it every day to see my baby’s progress which is like the highlight of my day… instead of it just being like ‘oh I’m quitting smoking, quitting smoking, quitting smoking’ it’s like you get to see sort of that, the positive side of it*” [IV05 pregnant, abstinent]    *“Personalising…even after pregnancy…like some breast-feeding smoking support as well because...I think all stuff like that will be really beneficial for new mums”* [IV14 pregnant, abstinent] |
|  | **Disadvantages**   - Unnecessarily duplication of information already provided by pregnancy apps - Individual preference to have apps clearly delineated by purpose | *I don’t think I would really want to see a pregnancy thing on my Stop Smoking app. That’s just my personal opinion, I do think it’s a good idea but for me personally no, I want to see pregnancy stuff on the pregnancy app and the stop smoking stuff on the stop smoking app. Because kind of like are you trying to make me feel bad, I feel bad enough.* [IV25 pregnant, abstinent] |
| **Badges and Rewards (e.g. for: quitting progress; engaging with different parts of an app/ website)** | | |
|  | **Advantages**   - Acknowledgement of progress – taking pride in achievement, earning virtual rewards - Fosters social comparison, competitiveness, engaging partners and significant others. - Utilisation of small tangible rewards may still be incentivising. | *“If you could do it with like your friends and family then I think it gives you a bit of a challenge, it makes it a bit of a competition which I really like”* [IV07 pregnant, abstinent]    *“Send me something physical or something that I can show my kids…that would keep me motivated...if you send me a little piece of paper that says ‘well done you’ve whatever’ or postcard or something...it makes it more real”* [IV25 pregnant, abstinent] |
|  | **Disadvantages**   - A patronising tone when congratulating achievements is discouraging - Virtual badges can be viewed as childish or as an ‘empty gesture’ - Potential for ‘fraudulent’ use, if utilising financial/voucher incentives | *“It does remind me of like the kid’s reward chart and as an adult I don’t need that as an incentive to quit”* [IV24 pregnant, abstinent]    *I think virtual [rewards] is better because I think if there was like, or maybe free nappies or free something for the baby or for yourself, it’s like if I don’t get it I’d be like ‘aww’ I would end up lying…. I think it would encourage cheating.* [IV23 pregnant, abstinent] ] |
| **Daily missions or tasks** | | |
|  | **Advantages**   - Provides motivation and reason to engage regularly with the app - Provides a positive, fun, focus during a challenging period - Reinforces a sense of accomplishment | *“It’s nice to have something to focus on when there’s no smoking”* [IV12 pregnant, smoking]    *“Just mental fun, with goals, achievable goals… like having a daily task or mission you can feel like you’ve accomplished something, I like the idea…break into some feel good things”* [IV17 pregnant, smoking] |
|  | **Disadvantages**   - Preference to be more autonomous - Novelty wears off, disengagement | *“I don’t think I would like to do that just because I think it would be a bit too much you know to do every week if you’ve got daily tasks and stuff…I think that would be overwhelming for me to be committed to do it every week”* [IVO6 pregnant, smoking]    *“I think I would start off using it and I’d be really energetic about it and then after a while it would become a chore and then I’d be like ‘I can’t be bothered to do that today*”[IV13 pregnant, abstinent] ] |
| **Notifications and prompts** | | |
|  | **Advantages**   - Facilitates engagement and action: prompt to visit the app and input information - Provides a sense of support – ‘feeling you are not alone’ - Convenience and immediacy when compared to standard interpersonal support - Can be tailored to the individual - timing and frequency of notifications, messages that reflect individual quit methods and journey | *“If you just have to log in yourself and look at sort of reminders of it then it’s not really something you kind of tend to choose to do I suppose, if it pops up on your phone then that’s different but you don’t really go ‘I’m just going to have a look at you know reasons why I shouldn’t smoke!’… But if I have to do like a CO2 reading every couple of days or something or whatever and then it comes up and says ‘Well done’ or ‘You’re not doing so well this week don’t forget that you know if you smoke then dah dah dah this happens”* [IV12 pregnant, smoking]]    *“An every day thing or maybe it’s a weekly thing but it’s there so even if your mind is not, you’ve forgotten about it if you see that notification pop up it makes you go into the app anyway”* [IV16 pregnant, smoking] |
|  | **Disadvantages**   - Frequent notifications can be overwhelming and/or irritating - Concerns about privacy: visibility of notifications and disclosure of pregnancy or smoking status - Potential for waning engagement, can be easily ignored - Fear that notifications may trigger cravings | *“It kind of just, it puts a negative on it you know, like it’s supposed to be something that you’re doing that’s positive and it kind of feels a little bit more like hard work because it’s constantly, you know like at the beginning when I was first with the Smoking Service it was quite regular contact at the beginning and it almost got a little bit too … you don’t want to have to keep having the same conversation or messages day in and day out of ‘Remember not to smoke’ and you’re like ‘I know, I’m trying!’ you know sometimes it gets a little bit, it’s a little bit too heavy”* [IV04 postpartum, abstinent]    *“[Stop smoking notifications] were quite regular and… quite sort of public about doing it… sometimes it’s something that’s quite private to you….like if you’re sitting out having dinner and your phone goes off everybody on the table like your phone lights up there like ‘oh what’s that?’ kind of thing”.* [IV15 pregnant, smoking] |
| **Tracking progress** | | |
|  | **Advantages**   - Highly motivational and helps foster a sense of achievement - Tracking financial savings made is incentivising, particularly when planning for a new baby - Regularity and continuity of monitoring and feedback - Ability to view visual representation of progress - Autonomy – a sense of control over quit attempt | *“Definitely a really, really good incentive for obviously somebody that’s given up smoking to see that ‘Oh I’ve gone five days, nine days’ do you know what I mean, it shows that they can do it”* [IV19 postpartum, abstinent]    *“One of the first things I did when I first found out I was pregnant was I sat down and worked out actually how much money I’d be able to save by not smoking…so yeah I think that [savings tracker] would be really useful”* [IV04 postpartum, abstinent]  *“Being able to see your progress is quite motivating as opposed to just being told every month or so ‘Yeah okay you’re doing well’ it’s good to kind of have it just something that you’re looking at*” [IV04 postpartum, abstinent]    *“I’m quite a visual person so for me that would be really useful, it gives you that sense of achievement doesn’t it to think ‘Oh I’m on day nine that’s really good’* [IV13 pregnant, abstinent]    *“So many times when I wanted to have a fag when I was stressed but I just said to myself ‘if I have a fag I’ve got to tell the app’ and all of my little stats get reset so it was more of like a little thing like that which is what helped me the most, because I had to tell the app I felt like every time I told the app I was in trouble*!” [IV14 pregnant, abstinent] |
|  | **Disadvantages**   - Doubts about sustaining engagement - Requires time and effort (in context of busy lives) - Requires honest appraisal and input of smoking behaviour | “*I can see myself giving that a go but whether I would actually remember?”* [IV10 postpartum, smoking]    *“For that to work you would have to really find a way to be honest and work out how many in the first place do you know what I mean and then you can really track your progress… it’s quite difficult to be like honest with yourself in the first place”.* [IV12 pregnant, smoking] |
| **Online forum** | | |
|  | **Advantages**   - Share experience with others who understand - Ability to be honest, less fear of being judged - Ability to share experience anonymously | *“So you can like chat to other mums and where there’s no risk of judgement from anyone…I find when I’m speaking to advisors and stuff some of them have never smoked or some of them don’t have children so they can’t actually relate to me and don’t understand why it’s hard”* [IV09 pregnant, smoking]    *“I think I would have liked to have more of like say a forum or a group chat to see how other people were getting on as well because I kind of felt quite alone with being pregnant and smoking and not wanting to talk about it to other people because of being ashamed and everything”* [IV22 postpartum, smoking]] |
|  | **Disadvantages**   - Potential for misinformation  and negative interaction, requirement for moderation - Hesitance to actively post - Forums with little active engagement are off-putting | *You just have no control over what people write I suppose [compared to} a professional service and quite an important part of being pregnant where you know only correct information is really the best … you do see stuff on there and you just think ‘Oh really!*’ [IV12 pregnant, smoking]    *“I just look at the messages and I think ‘Oh what a load of drips’…like ‘Just shut up!’  I think that’s what I would end up being like*” [IV13, pregnant, abstinent] |
| **Automated Chat bot** | | |
|  | **Advantages**   - Utility as a ‘first port of call’: potential to integrate escalation to ‘real person’ - Accessibility and immediacy of feedback - Anonymity can be preferable to real person interaction | *“To be able to chat to a robot as such just to get the sort of main information that you need for support and they can provide you with that then it’s going to be a lot less stressful than you scrolling through a page and stressing out because you can’t find something”* [IVO5 pregnant, abstinent]    *“If you had an option say of going for a chatbot and then you can’t get the answer you want so then going on to sort of real people…because you sort of went through every other avenue”* |
|  | **Disadvantages**   - Can be frustrating if unable to deal with user requests or needs, especially for more complex situations - Lack human contact or understanding | *“Quitting smoking is a very tough thing ...I struggle to see how, how a robot can get rid of my cravings it doesn’t make sense*” [IV07 pregnant, abstinent]    *“I think chatbots are useful depending on what the question is you are asking,… I think there are occasions where you do just want to speak to a real person”* [IV13 pregnant, abstinent] |
| **Access to an advisor (instant messaging, voice or video call)** | | |
|  | **Advantages**   - Human contact and understanding - Comfort and reassurance derived from knowledge that a real person is available (potentially 24/7) - Human accountability and empathy | *“That’s actually really great as well because then you’re not just dealing with an app with a problem, at least you know the person is a human at the end of it who actually will be talking to you as well”* [IV16 pregnant, smoking]    *“I think the human element of it just gives you that extra like motivation to do it, I guess if its fully automated...I personally would be a lot less inclined to do it”* [IV12 pregnant, smoking]    *“In the night and you’re really craving a cigarette and you’re sat on your own and sometimes you just need someone to talk to”* [IV02]    *“I needed my initial consultation, I needed to meet my Advisor to know that I had somebody there”* [IV07 pregnant, abstinent] |
|  | **Disadvantages**   - Lack of relationship-based support or consistency (different advisers) | *“The only thing I would worry about is if potentially like my [SSS} advisor was giving me different advice and then I’d be like ‘Well what do you actually listen to?”* [IV09 pregnant, smoking]    *“I think that it would depend if you got the same person each time because if you kind of build up a relationship I think there’s nothing worse than having to explain it all over again to somebody new*” [IV13 pregnant, abstinent] |
| **Remote CO monitoring** | | |
|  | **Advantages**   - More accessible and convenient of having a personal monitor - Reinforcement and validation of achievement - Sense of autonomy and self-management - Immediacy of feedback - Appeal of a gadget - Increasing knowledge of impact of smoking through visible indicator of changing CO levels - Engaging partners and significant others by sharing tools | *“You don’t have to actually go out of your house…you can do it when you want to so you know you’re not just booking an appointment for a certain time, if you want to do it you know just before bed it can get you into a habit of doing it”* [IVO6]    *“Everyone loves a gadget don’t they”* [IV17 pregnant, smoking]    *“I was like really quite amazed by it because I thought wow like you don’t actually even realise you’re literally breathing out toxic gas as such just by smoking fags…*[*my family] have all been like ‘Oh if I had one of them I would have quit smoking a lot quicker and a lot easier’ so if you did have the option of sharing them I think a lot of families would literally all quit smoking very easily*”  [IVO5 pregnant, abstinent] |
|  | **Disadvantages**   - Potential to ‘cheat’ – deliberately take false readings - Potential for inaccurate readings or technical problems | *“If you don’t smoke for one day you will come up as a non-smoker’s reading… I always found as long as I didn’t smoke the day I had a midwife appointment she wasn’t going to know, so I think with stuff like that I think it’s just a bit of a waste of money”* [IV14 pregnant, abstinent] |
| **NRT and e-cigarettes:  advice on and electronic provision of free nicotine replacement therapy (NRT), and potentially vapes, without interpersonal support** | | |
|  | **Advantages**   - More accessible and convenient to digitally order products and have home delivery - Digital modes could help facilitate NRT adherence - Potential utility to address barriers to uptake (e.g. reducing stigma around approaching a pharmacist or HCP) - Role for digital support to address misconceptions around e-cigarette use and safety by providing consistent information | *“I’ve got an Advisor now but I wouldn’t contact her before my appointment, so if I had an app it would…apps give you the information on there without delay and possibly get the Nicorette replacements to you quicker”* [IV07 pregnant, abstinent].    *“As long as you could try different things and you weren’t just set on it once you chose one because how do you know that one is going to work, you don’t, so as long as you could try different things if that wasn’t working for you I think that would be, that would definitely be good”* [IV07 pregnant, abstinent]    *“One thing with the app that you could do…it reminds you to put a patch on”* [IV12 pregnant, smoking]    *“I think at the beginning it would be more helpful to have some samples for example one or two patches, a few chewing gums, maybe a disposable electronic cigarette just to have a few puffs to see how it works…give some samples so the people can see which one is more suitable*” [IV20 pregnant, smoking] |
|  | **Disadvantages**   - Potential manipulation of the system if there is no identity check - Preference to have a trained advisor overseeing use and for trouble shooting - Requires reassurance that the source of advice and delivery is reputable. | *“I think it would be great but again the only thing is you don’t want people to take the mick and…claim they smoke forty fags a day because then they’re going to get a load of free gum and give it to their mates which obviously is not a bad thing but you don’t want to be you know wasting NHS resources and stuff do you?”* [IV13 pregnant, abstinent]    *“In pregnancy yes, it needs to be someone more specialised…someone who can talk to you at least once to decide the things and…to talk through the options*” [IV20 pregnant, smoking] |
| **Welcome box -delivered through the letter box, (containing for eg: CO monitor, Nicotine gum, Information leaflets, Progress chart)** | | |
|  | **Advantages**   - Provides motivation and focus to start quitting journey - Receiving a box as an incentive to downloading an app - Potential incorporation of NRT samples or e-cigarette - Providing a tangible ‘in your hands’ element - Engaging and reaching partners and significant others – a sharable resource | *“If you left it around the house your partner might pick up and look at so it could be quite good if there are partners that smoked.  So you could have something in there about like how your partner can be supportive through your pregnancy if you’re not smoking and stuff like that*”  [IV07 pregnant, abstinent]    *“Just the fact that you get a little welcome feel, you know ‘welcome to your journey’ and it’s kind of, it’s a nice way to kind of start off your journey with the resources that you need*” [IV24 pregnant, abstinent]    *“That sort of starting boost of ‘oh actually they’ve given me something, they’ve given me something to try, I don’t feel on my own’*…Y*ou focus on what’s in that box and …bits and pieces like that I think would be a massive, massive benefit”* [IV15 pregnant, smoking]    *“I did try [ nicotine gum] and it was vile and things have probably changed quite a bit now it’s a long time ago but yeah it was disgusting… if I could try something that I wasn’t spending money on and I could actually try it before I invested and know that (a) it worked and (b) it didn’t taste like an old ashtray”.* [IV17 pregnant, smoking] |
|  | **Disadvantages**   - Potential to ‘put to one side’ - Concern about resource implications (cost to NHS) - Potential unnecessary duplication of digital resources - Concerns about privacy and discrete packaging | *“If you just get a box I think for some people they might put that box on one side and think ‘right I’ll start that tomorrow”* [IV05 pregnant, abstinent]    “I *think also making the packaging of the parcel private….some people they may not want to tell their partner they’re trying Nicotine Replacement Therapy, they may just say ‘oh I’ve quit’ but they’re actually using a spray,  some people are like that they just, they prefer to keep that to themselves or if they may live with family, like if I still lived with my family my family never knew I smoked so if I wanted it I couldn’t actually get it delivered to my parents’ house because they’d be like ‘Well why do you need this?’* [IV15 pregnant, smoking] |
